# Supplementary figures and images for: Psychometric properties of ohip-edent b&h for conventional complete denture wearers
Source: PLoS One. 2023 Jan 20;18(1):e0280012. doi: 10.1371/journal.pone.0280012 (PMC9858044; doi:10.1371/journal.pone.0280012)

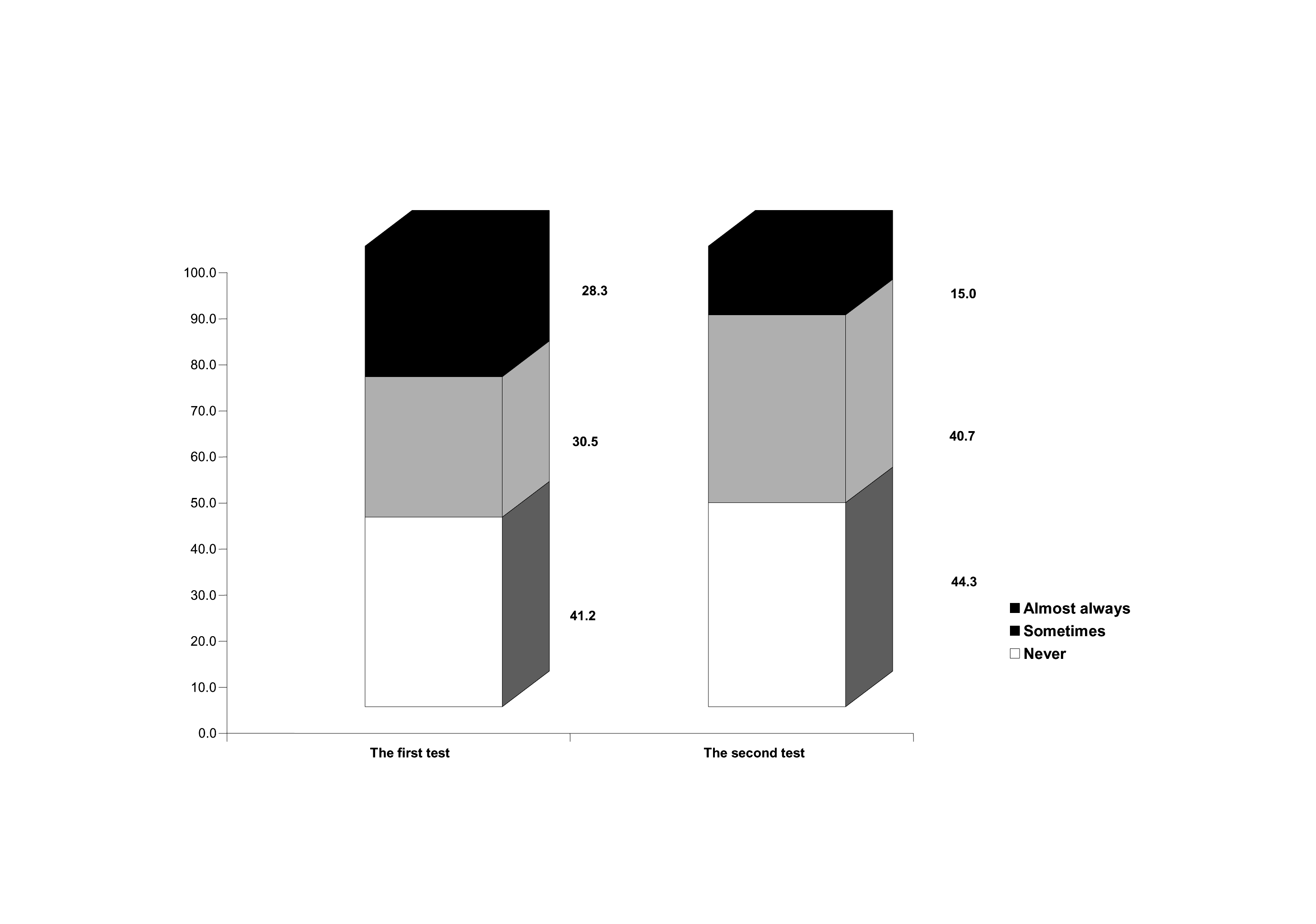

Supplement: S1 Fig — (TIF) [file pone.0280012.s001.tif]

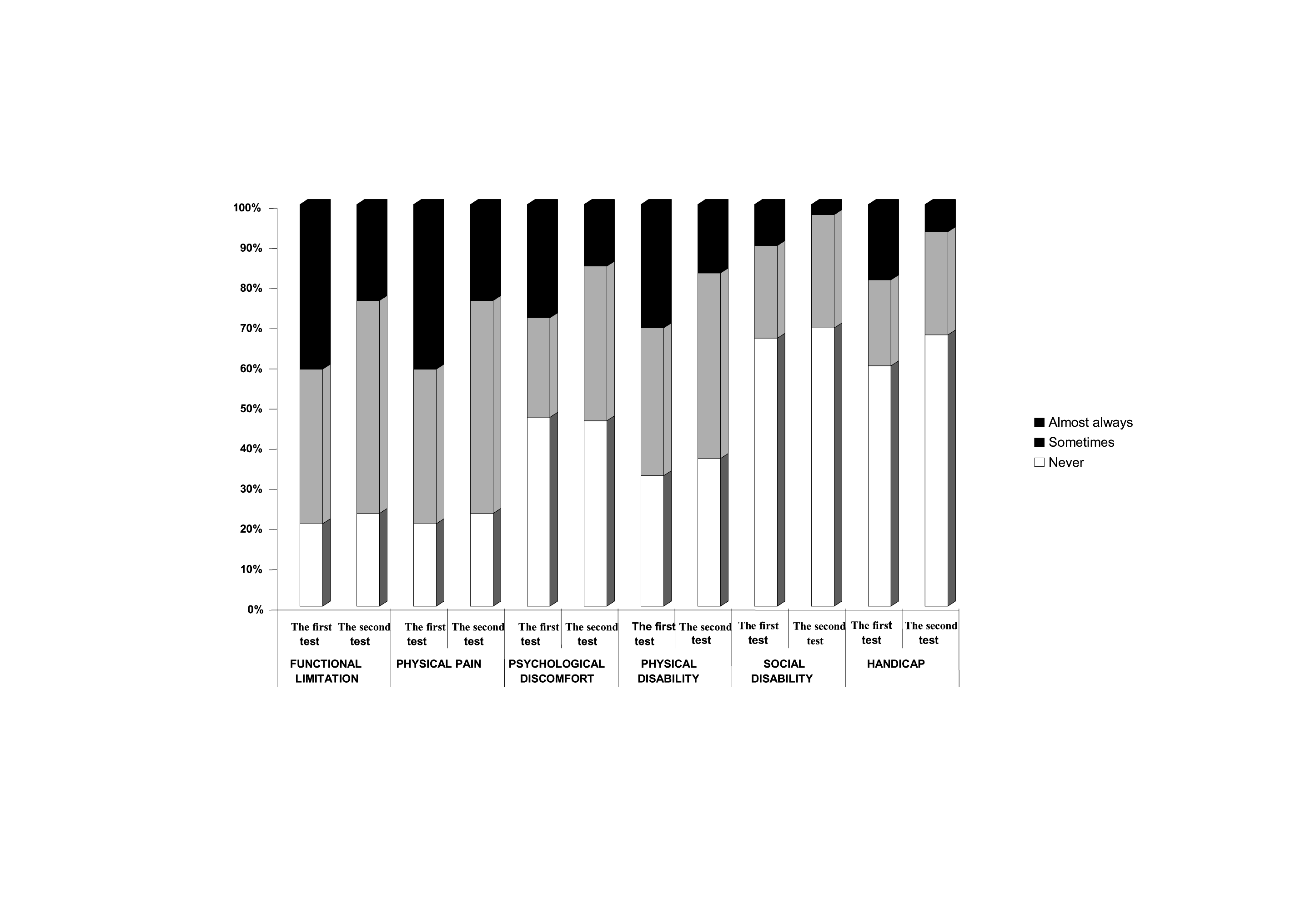

Supplement: S2 Fig — *T-test for equality of means. (TIF) [file pone.0280012.s002.tif]
