# Supplementary material for: Psychometric properties of ohip-edent b&h for conventional complete denture wearers
Source: PLoS One. 2023 Jan 20;18(1):e0280012. doi: 10.1371/journal.pone.0280012 (PMC9858044; doi:10.1371/journal.pone.0280012)
Supplement: S1 Table — ** = statistically high significance. (DOCX) [file pone.0280012.s003.docx]

Table1. Correlation of OHIP-EDENT-B&H summary score and all of domains of this score respecting the assessments of complete dentures before intervention by a specialist of prosthodontics

| Before intervention (denture corrections, relining, adjustments) | | OHIP-EDENT-B&H |
| --- | --- | --- |
| OHIP-EDENT Summary score |  |  |
|  | Sig. (two-tailed) |  |
|  | N | 117 |
| Assessment of denture acrylic teeth | Pearson correlation | 0.491^**^ |
|  | Sig. (two-tailed) | 0.000 |
|  | N | 117 |
| Assessment of a denture in general | Pearson correlation | 0.548^**^ |
|  | Sig. (two-tailed) | 0.000 |
|  | N | 117 |
| Assessment of denture’s basal part adaptation | Pearson correlation | 0.593^**^ |
|  | Sig. (two-tailed) | 0.000 |
|  | N | 117 |
| Assessment of denture occlusion | Pearson correlation | 0.509^**^ |
|  | Sig. (two-tailed) | 0.000 |
|  | N | 117 |
| Assessment of Functional equilibrium | Pearson correlation | 0.493^**^ |
|  | Sig. (two-tailed) | 0.000 |
|  | N | 117 |
| Assessment of Denture’s Retention | Pearson correlation | 0.487^**^ |
|  | Sig. (two-tailed) | 0.000 |
|  | N | 117 |
| Assessment of Denture’s Stability | Pearson correlation | 0.491^**^ |
|  | Sig. (two-tailed) | 0.000 |
|  | N | 117 |

**=statistically high significance
