# Supplementary material for: Psychometric properties of ohip-edent b&h for conventional complete denture wearers
Source: PLoS One. 2023 Jan 20;18(1):e0280012. doi: 10.1371/journal.pone.0280012 (PMC9858044; doi:10.1371/journal.pone.0280012)
Supplement: S2 Table — (DOCX) [file pone.0280012.s004.docx]

Table 2. The summary scores and scores of each domain ofthe OHIP-EDENT- B&H before and after corrections, adjustment and/or relining of complete dentures.

|  | | N | Mean | Standard deviation | Minimum | Maximum | Sig. (two-tailed)* |
| --- | --- | --- | --- | --- | --- | --- | --- |
| OHIP-EDENT | Before | 117 | 35.367 | 10.132 | 13.00 | 57.00 | 0.027 |
|  | After | 117 | 32.709 | 7.967 | 19.00 | 56.00 |  |
| Functional limitation | Before | 117 | 6.589 | 1.943 | 1.00 | 9.00 | 0.019 |
|  | After | 117 | 6.042 | 1.652 | 3.00 | 9.00 |  |
| Physical pain | Before | 117 | 7.863 | 2.392 | 2.00 | 12.00 | 0.003 |
|  | After | 117 | 7.401 | 2.042 | 4.00 | 12.00 |  |
| Psychological discomfort | Before | 117 | 3.803 | 1.475 | 2.00 | 6.00 | 0.010 |
|  | After | 117 | 3.418 | 1.219 | 2.00 | 6.00 |  |
| Physical disability | Before | 117 | 5.957 | 2.035 | 2.00 | 9.00 | 0.072 |
|  | After | 117 | 5.470 | 1.724 | 3.00 | 9.00 |  |
| Psychological disability | Before | 117 | 3.461 | 1.429 | 1.00 | 6.00 | 0.067 |
|  | After | 117 | 3.188 | 1.121 | 2.00 | 6.00 |  |
| Social disability | Before | 117 | 4.265 | 1.858 | 3.00 | 9.00 | 0.060 |
|  | After | 117 | 4.025 | 1.458 | 3.00 | 9.00 |  |
| Handicap | Before | 117 | 3.427 | 1.226 | 1.00 | 6.00 | 0.041 |
|  | After | 117 | 3.162 | 1.041 | 2.00 | 6.00 |  |

*T-test for equality of means
