# Supplementary material for: Psychometric properties of ohip-edent b&h for conventional complete denture wearers
Source: PLoS One. 2023 Jan 20;18(1):e0280012. doi: 10.1371/journal.pone.0280012 (PMC9858044; doi:10.1371/journal.pone.0280012)
Supplement: S1 File — Original source; Table 1A. (OHIP-EDENT-B and H)-domestic version of the influence of the specific components to oral status and oral health of edentulous patients which participated in the current study of SD Postic; additional questions are included in this table; Table 1B–Translations of questions in Table 1 from domestic language to English language and extension added for the purpose of the current study of SD Poštić. (PDF) [file pone.0280012.s005.pdf]

S1 Original source with translation to domestic regional language

Original source

OHIP EDENT, ALLEN LOCKER 2002

| Item | Domains | Item contains |
|---|---|---|
| 1 | Functional limitations | Trouble pronouncing words |
| 2 | | Taste worse |
| 3 | Physical pain | Painful aching |
| 4 | | Uncomfortable to eat |
| 5 | Psyhological discomfort | Self-concious |
| 6 | | Tense |
| 7 | Physical disability | Diet unsatisfactory |
| 8 | | Interrupt meals |
| 9 | Psychological disability | Difficult to relax |
| 10 | | Been embarrassed |
| 11 | Social disability | Irritable with others |
| 12 | | Difficulty doing jobs |
| 13 | Handicap | Life unsatisfying |
| 14 | | Unable to function |

Table 1A. (OHIP-EDENT-BandH)-domestic version of the influence of the specific components to oral status and oral health of edentulous patients which participated in the current study of SD Postic; additional questions are included in this table.

Subjekti su odgovarali na pitanja koja se tiču učestalosti uticaja oralnih aspekata zdravlja na dnevne aktivnosti, korišćenjem lestvice: 0- nikada, 1- gotovo nikada, 2- vrlo često.

FUNKCIONALNO OGRANIČENJE

1. Da li imate smetnje pri žvakanju bilo kakve hrane?
2. Da li vam se hrana zadržava na zubima ili protezi?
3. Da li osećate da vam proteza ne naleže kako treba?

FIZIČKI BOL

4. Da li imate smetnje pri žvakanju bilo kakve hrane?
5. Da li vam se hrana zadržava na zubima ili protezi?
6. Da li osećate da vam proteza ne naleže kako treba?
7. Osećate li bol u ustima?

PSIHOLOŠKA NELAGODNOST

8. Da li imate smetnje pri žvakanju bilo kakve hrane?
9. Da li vam se hrana zadržava na zubima ili protezi?

FIZIČKA INVALIDNOST

10. Da li osećate da vam proteza ne naleže kako treba?
11. Osećate li bol u ustima?
12. Da li osećate nelagodnost zbog problema u vašim ustima?

PSIHOLOŠKA INVALIDNOST

13. Da li ste uznemireni zbog problema sa svojim zubima, ustima ili protezom?
14. Da li ste bili malo uzrujani zbog problema sa svojim zubima, ustima ili protezom?

SOCIJALNA INVALIDNOST

15. Da li ste izbegavali da izlazite napolje u spoljnu sredinu zbog roblema sa svojim zubima, ustima ili protezom?
16. Da li ste bili manje uviđavni prema porodici zbog vašig objektivnih problema sa svojimzubima, ustima ili protezom?
17. Da li ste bili pomalo napeti prema ljudima iz okruženje zbog problema sa svojim zubima, ustima ili protezom?

HENDIKEP

18. Da li ste se osećali nesposobnim da uživate u društvu drugih ljudi zbog problema sa

| | |
|---|---|
| | svojim zubima, ustima ili protezom? |
| 19. | Da li ste se uopšte osećali da je vaš život manje kvalitetan  zbog problema sa svojim zubima, ustima ili protezom? |

Table 1B – Translations of questions in table 1 from domestic language to English language and extension added for the purpose of the current study of SD Poštić

Functional limitation

1- Have you had difficulty chewing any foods because of problems with your teeth, mouth or dentures?

2- Have you had food catching in your teeth or dentures?

3- Have you felt that your dentures have not been fitting properly?

Physical pain

4- Have you had painful aching in your mouth?

5- Have you found it uncomfortable to eat any foods because of problems with your teeth, mouth or dentures?

6- Have you had sore spots in your mouth?

7- Have you had uncomfortable dentures?

Psychological discomfort

8- Have you been worried by dental problems?

9- Have you been self conscious because of your teeth, mouth or dentures?

Physical disability

10- Have you had to avoid eating some foods because of problems with your teeth, mouth or dentures?

11- Have you been unable to eat with your dentures because of problems with them?

12- Have you had to interrupt meals because of problems with your teeth, mouth or dentures?

Psychological disability

13- Have you been upset because of problems with your teeth, mouth or dentures?

14- Have you been a bit embarrassed because of problems with your teeth, mouth or dentures?

Social disability

15- Have you avoided going out because of problems with your teeth, mouth or dentures?

16- Have you been less tolerant of your spouse or family because of problems with your teeth, mouth or dentures?

17- Have you been a bit irritable with other people because of problems with your teeth, mouth or dentures?

Handicap

18- Have you been unable to enjoy other people's company as much because of problems with your teeth, mouth or dentures?

19- Have you felt that life in general was less satisfying because of problems with your teeth, mouth or dentures?
