# Supplementary material for: Psychometric properties of ohip-edent b&h for conventional complete denture wearers
Source: PLoS One. 2023 Jan 20;18(1):e0280012. doi: 10.1371/journal.pone.0280012 (PMC9858044; doi:10.1371/journal.pone.0280012)
Supplement: Attachment — Submitted filename: Response to Reviewers-SDP-07Nov22.docx [file pone.0280012.s006.docx]

Dear Plos One,

I finished revision of the previous version of the manuscript.

I seriously tried to follow demands of the reviewers, and because of that I truly hope that as of professional integrity of this manuscript after my revision no needs for additional changes should be made. However, considering the English language I must admit how professional court interpreter and translator for the English language conjointly with the Professor for English language in the University School of Dental medicine in Beograd reviewed this changed text of the manuscript, but they have not official dental-professional competences with certificates for editing. Because of that, if we were not successful in spelling, grammar and corrections I could additionally proceed this text, altogether with reviewers’ comments to Elsevier Editing service (which I use before) or some other editing professional service which you suggest. In this case, however I should ask you for much more time and postponed deadline for corrections considering the fact that professional editing services need some extended time to analyze and change the text of the manuscript.

Journal Requirements:

When submitting your revision, we need you to address these additional requirements.

1. Please ensure that your manuscript meets PLOS ONE's style requirements, including those for file naming. The PLOS ONE style templates can be found at <https://journals.plos.org/plosone/s/file?id=wjVg/PLOSOne_formatting_sample_main_body.pdf> and 
<https://journals.plos.org/plosone/s/file?id=ba62/PLOSOne_formatting_sample_title_authors_affiliations.pdf>

**Author response: The paper is formatted according to the requirements of the journal.**

2. We note that you have stated that you will provide repository information for your data at acceptance. Should your manuscript be accepted for publication, we will hold it until you provide the relevant accession numbers or DOIs necessary to access your data. If you wish to make changes to your Data Availability statement, please describe these changes in your cover letter and we will update your Data Availability statement to reflect the information you provide

**Author response: All information related to the data in the paper can be obtained upon request from the author. Data are not available in other electronic databases.**

3. Thank you for stating the following in your Competing Interests section:

"No authors have competing interest"

Please complete your Competing Interests on the online submission form to state any Competing Interests. If you have no competing interests, please state "The authors have declared that no competing interests exist.", as detailed online in our guide for authors at <http://journals.plos.org/plosone/s/submit-now> 
 This information should be included in your cover letter; we will change the online submission form on your behalf

**Author response: The authors have declared that no competing interests exist.**

4. Thank you for stating the following financial disclosure:

"No authors have competing interest."

At this time, please address the following queries:

a) Please clarify the sources of funding (financial or material support) for your study. List the grants or organizations that supported your study, including funding received from your institution. 
b) State what role the funders took in the study. If the funders had no role in your study, please state: “The funders had no role in study design, data collection and analysis, decision to publish, or preparation of the manuscript.”
c) If any authors received a salary from any of your funders, please state which authors and which funders.
d) If you did not receive any funding for this study, please state: “The authors received no specific funding for this work.”

Please include your amended statements within your cover letter; we will change the online submission form on your behalf.

Please review your reference list to ensure that it is complete and correct. If you have cited papers that have been retracted, please include the rationale for doing so in the manuscript text, or remove these references and replace them with relevant current references. Any changes to the reference list should be mentioned in the rebuttal letter that accompanies your revised manuscript. If you need to cite a retracted article, indicate the article’s retracted status in the References list and also include a citation and full reference for the retraction notice.

**Author response: The funders had no role in study design, data collection and analysis, decision to publish, or preparation of the manuscript. The authors received no specific funding for this work.**


Reviewers' comments:

Reviewer's Responses to Questions

Comments to the Author

1. Is the manuscript technically sound, and do the data support the conclusions?

The manuscript must describe a technically sound piece of scientific research with data that supports the conclusions. Experiments must have been conducted rigorously, with appropriate controls, replication, and sample sizes. The conclusions must be drawn appropriately based on the data presented.

Reviewer #1: Partly

Reviewer #2: Yes

**Author response: Thank you very much for your comments, the paper is now prepared according to the technical requirements of the journal.**

2. Has the statistical analysis been performed appropriately and rigorously?

Reviewer #1: Yes

Reviewer #2: Yes

**Author response: Thank you very much for your comments.**

3. Have the authors made all data underlying the findings in their manuscript fully available?

The [PLOS Data policy](http://www.plosone.org/static/policies.action#sharing) requires authors to make all data underlying the findings described in their manuscript fully available without restriction, with rare exception (please refer to the Data Availability Statement in the manuscript PDF file). The data should be provided as part of the manuscript or its supporting information, or deposited to a public repository. For example, in addition to summary statistics, the data points behind means, medians and variance measures should be available. If there are restrictions on publicly sharing data—e.g. participant privacy or use of data from a third party—those must be specified.

Reviewer #1: No

Reviewer #2: Yes

**Author response: Thank you very much for your comments.**

4. Is the manuscript presented in an intelligible fashion and written in standard English?

PLOS ONE does not copyedit accepted manuscripts, so the language in submitted articles must be clear, correct, and unambiguous. Any typographical or grammatical errors should be corrected at revision, so please note any specific errors here.

Reviewer #1: Yes

Reviewer #2: No

**Author response: Thank you very much for your comments, now the paper was reviewed by a native English teacher.**

5. Review Comments to the Author

Reviewer #1: Introduction
I understand that being direct in the proposal is good for the manuscript, however, it lacks a better foundation! I suggest that the Introduction be revised and expanded.

**Author response: Thank you very much for your comments. We have significantly expanded the introduction regarding the importance of these scales for assessing quality of life based on oral health assessment.**

Methods
OK, but the description is a little confusing. I suggest a review of the English for better suitability.

**Author response: Thank you very much for your comments, now the paper was reviewed by a native English teacher.**

Results
4th paragraph: 39,33% indicated the prostheses as excellent and 78.67% as good. Added together this exceeds 100%!!! Please check!

**Author response: Thank you very much for your comments, we corrected the mistake, it was excellent with 39.33% and good with 60.67%.**

Limitations
Wouldn't this be important information in the Methods?

**Author response: Thank you for your comments. The limitations of this study are noted and explained in the end of the first paragraph of Material and methods section after authors’ revision.**

Discussion
Well conducted.

**Author response: Thank you very much for your comments.**

Conclusion

**Author response: Thank you very much for your comments.**
OK

Tables, Figures (graphs) and Supporting Information
Ok

**Author response: Thank you very much for your comments.**

Reviewer #2: Kindly note the following:
ABSTRACT: The methods section is extremely deficient and needs more information.
INTRODUCTION: This section is very short and does not give sufficient background about the topic under study.
Additionally, a thorough grammatical and spelling check-up throughout the whole manuscript is requested.

**Author response: Thank you very much for your comments. We have significantly expanded the introduction regarding the importance of these scales for assessing quality of life based on oral health assessment.**

**Also, we have significantly expanded the abstract.**

**Author response: Thank you very much for your comments, now the paper was reviewed by a native English teacher**
